# Stealing the Show: KSHV Hijacks Host RNA Regulatory Pathways to Promote Infection

**DOI:** 10.3390/v12091024

**Published:** 2020-09-14

**Authors:** Daniel Macveigh-Fierro, William Rodriguez, Jacob Miles, Mandy Muller

**Affiliations:** Department of Microbiology, University of Massachusetts, Amherst, MA 01003, USA; dmacveighfie@umass.edu (D.M.-F.); williamrodri@umass.edu (W.R.); jmmiles@umass.edu (J.M.)

**Keywords:** KSHV, RNA regulation, m6A, SOX, G4s, miRNA, circRNA

## Abstract

Kaposi’s sarcoma-associated herpesvirus (KSHV) induces life-long infections and has evolved many ways to exert extensive control over its host’s transcriptional and post-transcriptional machinery to gain better access to resources and dampened immune sensing. The hallmark of this takeover is how KSHV reshapes RNA fate both to control expression of its own gene but also that of its host. From the nucleus to the cytoplasm, control of RNA expression, localization, and decay is a process that is carefully tuned by a multitude of factors and that can adapt or react to rapid changes in the environment. Intriguingly, it appears that KSHV has found ways to co-opt each of these pathways for its own benefit. Here we provide a comprehensive review of recent work in this area and in particular recent advances on the post-transcriptional modifications front. Overall, this review highlights the myriad of ways KSHV uses to control RNA fate and gathers novel insights gained from the past decade of research at the interface of RNA biology and the field of KSHV research.

## 1. Introduction

Kaposi’s sarcoma-associated herpesvirus (KSHV), also known as human herpesvirus-8 (HHV-8) is a γ2-herpesvirus that is the causative agent of Kaposi’s sarcoma (KS), KSHV inflammatory cytokine syndrome (KICS), primary effusion lymphoma (PEL), and a plasmablastic form of multicentric Castleman’s disease (MCD) [1,2,3]. Although KSHV and other oncoviruses such as Epstein-Barr virus (EBV) are powerful carcinogens, most people infected are asymptomatic. KSHV infection can be either latent or lytic: during latency, KSHV only expresses a few genes involved in viral persistence and host immune evasion [4]; however, upon reactivation by environmental and/or physiological stimuli, KSHV re-enters its lytic phase where the viral genome is amplified and new virions are formed [5]. The latent stage of KSHV life cycle can last for decades, but once in its lytic form, KSHV is particularly effective at exploiting the host gene expression machinery for its own benefit. In particular, KSHV benefits from a long co-evolution history with its host, and from a large viral genome which houses many viral genes and a number of regulatory elements like viral microRNAs (miRNAs), long non-coding RNAs (lncRNAs), and circular RNAs (circRNAs) [6,7,8]. Taken together, these characteristics make KSHV a powerful manipulator of the host gene expression environment. 

**Figure 1:**
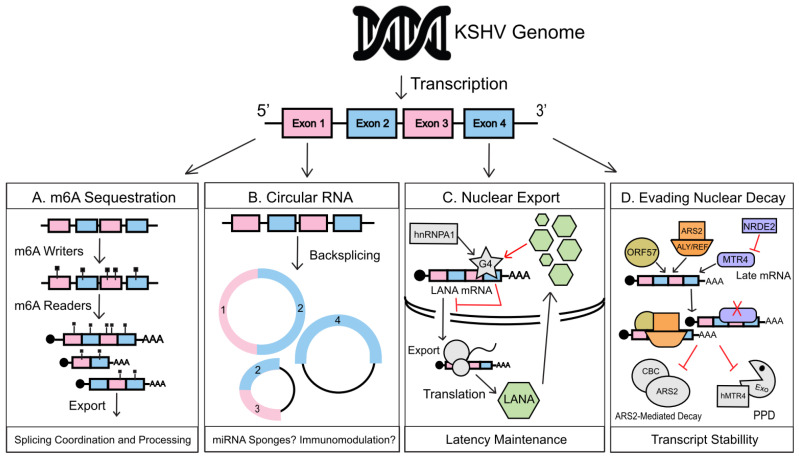
Kaposi’s sarcoma-associated herpesvirus (KSHV) manipulation of nuclear RNA regulation. (**A**) During lytic reactivation, KSHV transcripts are preferentially m^6^A modified over host RNAs, co-opting host m^6^a readers and writers to direct viral mRNA post-transcriptional regulation, splice patterns, and nuclear export. (**B**) Back-splicing of viral RNAs leads to the production of viral circular RNAs (circRNAs). Viral circRNAs could act as microRNA (miRNA) sponges to enhance translation of their linear counterparts and potentially encode novel viral peptides with as-yet to be defined functions. Several circRNAs are also packaged into KSHV virions, which points to a potential immunomodulatory role during primary infection. (**C**) G-quadruplexes are RNA secondary structures found within guanine rich regions of pre-mRNA formed by a unique hydrogen bond stacking interaction. A G4 structure within the LANA mRNA regulates hnRNPA1-facilitated transcript export. It also binds LANA protein in a negative feedback loop that finely tunes the amount of LANA transcripts within the cytoplasmic RNA pool. G4 regulation of LANA mRNA balances the amount of LANA being produced during KSHV latency, aiding in latency maintenance. (**D**) The lytic early gene, ORF57, binds to KSHV early and delayed early transcripts in complex with ALYREF and ARS2, protecting them from targeting by the 3′ PAPα/γ-mediated RNA decay (PPD) and Cap-Binding Complex/ARS2-mediated (CBC/ARS2) nuclear decay pathways. Late KSHV mRNAs when produced prematurely are targeted for PPD-based decay by cellular MTR4 contributing to temporal viral gene regulation. PPD-targeting of these mRNA, when no longer necessary, is subverted as nuclear speckles fuse with viral replication compartments, which allows nuclear RNAi-defective 2 (NRDE2) to restrict the interaction of MTR4 with late transcripts.

To hijack the host, KSHV triggers an intricate takeover of cellular RNA fate ranging from widespread manipulation of RNA decay to influencing patterns of RNA expression. By seizing control of RNA surveillance pathways, the virus can fine-tune the global gene expression environment throughout both phases of infection. This newly forged pro-viral RNA landscape allows KSHV unprecedented control over viral gene expression, which requires extensive temporal regulation and the capacity to widely remodel gene expression pathways. Many mechanisms are at play to allow the cell to maintain tight control over mRNA fate through mechanisms often associated with physical RNA features such as pre-mRNA splice patterns, 5′ cap, 3′ poly-A tail, and sequences/modifications that recruit trans-acting factors that alter mRNA stability. Post-transcriptional modifications and RNA editing also appear to be particularly important for KSHV-mediated takeover of the host. This review will explore the strategies evolved by KSHV to co-opt or subvert cellular RNA regulatory pathways that influence different stages of mRNA development during viral infection (Figure 1). Many of these targeted pathways, such as the N6-methyladenosine (m^6^A) modification, can regulate viral RNA stability and function at a global scale while other more specific features, such as G4 quadruplex motifs, can enforce strict control over viral RNA translation. There are also several virally encoded non-coding RNAs (ncRNA) that indirectly influence viral mRNA fate through a variety of mechanisms that mimic or even outperform their cellular counterparts during infection. We will also examine the regulation of key trans-acting viral proteins like ORF57 that protect viral RNAs from nuclear decay and the KSHV endoribonuclease, SOX, which triggers a massive RNA degradation event during lytic reactivation. KSHV’s capacity to take advantage of cellular RNA regulatory networks with such precision gives researchers a window into the never-ending arms race between viruses and their host. Through the work reviewed here, we wish to reveal research that shines a light on the strategies KSHV uses to enhance its viral gene expression. The studies illustrated here undoubtedly open new avenues in which cellular RNA fate and KSHV exploitation of those systems can be explored in the future. 

## 2. Nuclear Regulation of RNA during KSHV Infection

As the cell initiates RNA transcription, it also begins the process of regulating the fate of RNA very closely. As transcription occurs, RNA transcripts will undergo a series of maturation processes mainly consisting of co-transcriptional splicing, addition of a 5′ cap, and a 3′ poly-A tail. These processes are mediated by cellular factors that interact with one another and promote maturation of the RNA and ultimately determine their localization and function [9,10]. Another co-transcriptional maturation process is the addition of chemical modifications, and in the case of messenger RNA (mRNA), N6-methyladenosine (m^6^A). These chemical additions emerge as crucial for the regulation of many RNA processes, like splicing, translation, stability, and decay. Before RNAs can interact with nuclear export machinery, they must contend with mechanisms that regulate the amount of transcript that gets exported into the cytoplasm or nuclear decay pathways, like the CBCN-mediated RNA decay pathway, which protects the cell from aberrant transcripts. In this section, we will be reviewing how KSHV uses m^6^A to regulate splicing of its own transcripts, ORF57, to escape nuclear decay pathways and G-Quadruplexes on its mRNA to control nuclear export. Circular RNAs (circRNAs) will also be a new addition to the vast list of KSHV transcript regulator and will be discussed below.

### 2.1. Co-Transcriptional Regulation

M^6^A. The m^6^A machinery consists of a complex set of proteins that coordinate the regulation of RNA: there is a writer complex that includes METTL3, METTL14, WTAP, METTL16, RBM15, and KIAA1429 [11,12,13,14] and is responsible for adding a methyl group to a target adenine. The addition occurs in the nucleus during transcription and tends to be deposited on DRACH motifs [15,16]. m^6^A addition is a highly dynamic process that also includes a removal mechanism catalyzed by demethylases or erasers like FTO or ALKBH5 [17,18]. These chemical modifications are important coordinators of RNA regulation that KSHV has recently been shown to co-opt for control of viral mRNA. In work done by Tan et al., it was revealed that KSHV possesses m^6^A modifications both on latent and lytic viral transcripts. Intriguingly, during KSHV lytic reactivation, they also observed a general decrease of m6A deposition on cellular mRNAs [19]. In latently infected cells, hypomethylation at the 5′ end of cellular mRNAs and hypermethylation at the 3′ ends were observed compared to non-infected cells [19]. The disproportionate amount of methylation that is deposited on the cellular transcripts during infection raises the question of what is the purpose of these modifications? Increased amount of 3′ UTR m^6^As possibly allows for enhanced 3′ UTR regulation, which has been known to affect localization, translation initiation, and the stability/decay rate of mRNAs [20]. In fact, it was revealed that numerous pathways implicated in cellular transformation and oncogenic signaling are upregulated during infection in correlation with an increased amount of m^6^A methylation. One of the hypotheses raised by the authors is that the enrichment of methylation in the 3′ UTR on cellular transcripts may help mediate targeting of miRNAs by KSHV own miRNAs that are known to be essential for KSHV-induced cellular transformation [21]. However, further examinations did not find any correlation between differential methylation and targets of KSHV miRNAs [19]. All this research has helped prompt a deeper dive into how KSHV is affected by m^6^A modifications during infection and what occurs to the m^6^A landscape when KSHV switches from the latent to the lytic portion of its life cycle. Recent studies from Ye et al. in vivo and Hesser et al. in vitro have also reported that the amount of m^6^A-modified KSHV mRNA increases during lytic reactivation while decreasing on cellular mRNAs [22,23]. The decreased amount of cellular m^6^A deposition may be due to viral-induced widespread RNA decay leading to a decrease in available transcripts for m^6^A deposition and, therefore, an increase in viral transcript availability. Another possibility is KSHV influences m^6^A deposition directly by increasing viral RNA likelihood of being methylated or by affecting the m^6^A machinery itself. The counterpart of this shift in m^6^A deposition towards viral mRNA is that the reduced m^6^A deposition on cellular RNA could help dampen some host anti-viral processes by affecting host mRNA stability and/or translation. 

m^6^A modifications are decoded by a collection of cellular factors that “read” this modification and enact the associated function. Depending on the localization of the modification on the mRNA, different reader proteins will be recruited and the resulting effect on the modified RNA will differ [13,24,25,26]. Proteins with a YTH domain, like YTHDC1-2 and YTHDF1-3, have been characterized as major m^6^A readers that contain an aromatic cage that allows direct binding of the protein to the m^6^A modification [27]. One of these reader proteins, YTHDC1, often coordinates splicing events, epigenetic silencing, and nuclear export of mRNA [28,29,30]. Ye et al. showed that the functions of YTHDC1 activity are co-opted by KSHV: it was found that chemically removing m^6^A using 3-deazaadenosine (DAA)—an inhibitor of METTL3—a prominent m6A writer—prevented pre-mRNA splicing of the KSHV major lytic switch protein, RTA (ORF50), which resulted in a reduction of viral lytic replication [22]. Although there were multiple m^6^A sites found in the RTA pre-mRNA, it was revealed that a single methylation site in exon 2 was responsible for enhancing splicing by using its m^6^A site to recruit YTHDC1 which in turn recruits SRF3 and SRS10 to aid in splicing. However, RTA pre-mRNA is known to encompass many different splice variants, so it is possible that the other m^6^A sites found by Ye et al. interact with other m^6^A nuclear reader proteins such as hnRNP-C1/2 and hnRNP-A2B1 to regulate differential splicing. More recently, a new class of readers from the “Royal” family, specifically Staphylococcal nuclease domain-containing protein 1 (SND1), was shown to bind directly to m^6^A modifications similarly to YTH domain proteins [31]. It was discovered that when SND1 was knocked down, the amount of RTA RNA was reduced by 50%, suggesting that SND1 affects the stability of unspliced RTA RNA [31]. Given the extensive role of m^6^A in aiding the recruitment of splicing factors, uncovering more m^6^A readers on novel m^6^A sites will reveal a complex growing regulatory network of co-transcriptional regulators that will help us better understand cellular and KSHV RNA fate. 

KSHV Circular RNA. Circular RNAs (circRNAs) are a class of 3′ to 5′ cyclized RNAs that are the product of a unique form of precursor RNA processing known as back-splicing [32,33]. The process of back-splicing involves the joining of a 3′ splice donor site and an upstream 5′ splice acceptor to form a covalent linkage, generating a closed cyclized RNA molecule. These circular RNAs inherently lack poly-A tails and can be either short or long depending on the number of exons found within the linear mRNA counterpart. While these RNAs were initially considered oddities of abnormal splicing, several reports have found that they in-fact represent a significant percentage of the mammalian RNA pool [32]. Due to their circular structure and lack of 5′ and 3′ structural features, these RNAs are naturally resistant to targeting by cellular exonucleases and in many cases can accumulate to even higher levels than their cognate mRNAs [34]. Coupled with this enhanced stability, a growing list of molecular functions have been attributed to human circRNAs in a variety of biological systems including acting as miRNA sponges, splicing regulators, translation templates, and even serving as modulators of the immune response to viral infection [35,36,37,38,39]. However, several outstanding questions continue to surround this budding field including (i) what trans- and cis-acting RNA factors govern back-splicing efficiency and frequency? (ii) what molecular mechanisms drive recently observed circRNA phenotypes? and (iii) what are the advantages of circRNA production over linear RNA production in response to environmental stressors? Answers to these questions and more remain an active area of research in this exciting area of RNA biology. 

Recent large-scale screens have uncovered that virally encoded circRNAs are also expressed during latent and lytic KSHV infection. One of the first screens of the KSHV genome for circRNA expression was performed by Toptan et al. in 2018, uncovering several novel viral circRNAs, most abundantly derived from the vIRF4 and PAN/K7.3 gene loci. This same list also found several other low abundance circRNAs potentially derived from multiple lytic ORFs [40]. In parallel to this screen, using a circRNA-specific RNA-seq, Tagawa and colleagues expanded the list KSHV encoded circRNAs [41]. In the RTA-inducible BCBL-1 cell model, they found that viral circRNAs are more highly expressed during lytic reactivation and suggested they play roles in KSHV tumor progression including regulation of viral gene expression and host cell proliferation. Furthermore, they also demonstrated that viral circRNA expression varies between KSHV-infected cell lines and even clinical KS samples. Observations by each of these screens laid the foundation for the study of an entirely new class of KSHV-encoded ncRNAs. Work in this area will undoubtedly uncover novel cis and trans-acting RNA elements that direct the regulation and even functionality of circRNAs. Though this field is still in its infancy, several groups have already begun to tie the viral circRNAs to several pro-viral pathways including cell proliferation and even modulation of the anti-viral response to KSHV infection [41]. 

Many questions continue to surround the mechanisms that regulate the expression of KSHV-encoded circRNAs and their impact within the viral RNA pool. Several large-scale screens have already identified numerous small back-splice junctions throughout the KSHV genome, while curiously only a single major junction gives rise to one of the most abundant KSHV circRNAs, circ-vIRF4. For human circRNAs, cis factors such as inverted Alu elements within the nascent pre-RNA between the donor and acceptor sites have been shown to mediate their interaction leading to cyclization [32]. While such sequences have yet to be identified within the KSHV genome, it does not rule out the possibility that similar mechanisms could be orchestrated by smaller inverted sequences as suggested by previous reports [42]. Several cellular trans factors such as QKI (Quaking Homolog, KH Domain RNA Binding) and MBNL (Muscleblind-Like Splicing Regulator 1) have also been shown to promote back-splicing events but these factors among others have yet to be directly tied to the synthesis of KSHV circRNAs [43]. Further research in this arena should begin the work of building a definitive list of host and even viral factors that may regulate the frequency of viral back-splicing events and whether the composition of these players shift between the different phases of infection. In terms of coding capacity, none of the reported KSHV circRNAs have been associated with ribosome binding or contain internal ribosome entry sites (IRES). However, recent evidence suggests that other mechanisms such as cap-independent translation via m^6^A methylation could in fact drive the translation of these transcripts [44]. This is particularly interesting when we consider that many of these circRNAs contain exonic regions not typically encoded in their cognate mRNAs, which could lead to as-yet-described viral peptides [41,45]. Furthermore, the idea that these could be novel coding transcripts is complicated further when we consider the expression circPAN, whose linear counterpart is one of the most abundant non-coding RNAs found during lytic KSHV infection. Linear PAN does not contain introns or exons and, therefore, remains an oddity among the viral circRNAs as it remains unclear what drives the circPAN back-splicing event. The existence of circPAN also brings about several questions including (i) do these circular transcripts encode peptides unlike their linear counterparts? (ii) what host and viral factors regulate the production of circPAN, and (iii) do the cis- and trans- acting RNA elements of linear PAN continue to function in circPAN? Beyond their coding capacity, circRNAs could also act as a sponge for host miRNAs preventing them from influencing viral mRNAs. This model has been confirmed for an EBV circRNA, circRPMS1, where multiple human miRNAs including miR-31, 203, and 451 were found to bind to it, which induced an overall anti-apoptotic response in adherent EBV-infected cells [46]. To date, a similar role for the KSHV-encoded circRNAs has yet to be described but the possibility remains as the circRNAome continues to be uncovered. 

Like circ-PAN, circ-vIRF4 also represents a curious case in both its expression across various KSHV-infected cell types and the current air of ambiguity that surrounds its regulation between latency and lytic reactivation. Previous reports have shown that linear vIRF4 is expressed at low levels during latency and is highly expressed during lytic infection. Conversely, a recent report by Abere et al. found that circ-vIRF4 was expressed at lower levels than its cognate linear form during latency and strangely was only marginally enhanced or even reduced in most PEL cell lines following lytic reactivation [47]. While this could be a simple difference in lytic gene induction, a more intriguing possibility is that an entirely separate level of post-transcriptional regulation may govern biogenesis of circ-vIRF4 during the lytic phase apart from those that regulate circ-PAN and circ-K7.3. From this point, Abere et al. proceeded to investigate the localization of circRNAs using a highly sensitive in-situ hybridization strategy designed to detect the unique back-splice junctions of circRNAs known as BaseScope. Using this strategy, they demonstrated that circ-vIRF4 puncta were abundant across all tested KSHV-positive PEL cell lines. However, circ-vIRF4 puncta did not increase following lytic reactivation, in accordance with previous observations, which suggests that there may be a latency-associated regulatory role for this circRNA. One final area explored by Abere and colleagues was whether KSHV-encoded circRNA are incorporated into KSHV virions as had been previously described for other viral non-coding RNAs [47]. Surprisingly, they found that the smaller, roughly 530 nt, cytoplasmic isoform of circ-vIRF4 (circ-vIRF4.IR) and not its nuclear isoform was indeed packaged into the tegument of KSHV virions along with variations of circPAN and circK7.3. The distinction between the cytoplasmic and nuclear circ-vIRF4 incorporation may simply be attributed to differences in localization. However, the nuclear circ-vIRF4 does contain an intronic region from its linear cognate that may also play a part in the observed difference in incorporation. These observations raise several questions such as (i) are only cytoplasmic circRNA packaged into virions? (ii) Are KSHV circ-RNAs, carried from cell-to-cell by virions, immunogenic and/or immunomodulatory? and (iii) what cellular and/or viral machinery are involved in packaging of these circRNAs into virions?

The field of circRNA biology, having matured well over the past decade, has turned much of our understanding of post-transcriptional regulation and the contribution of splicing to control RNA fate on its head. The discovery of KSHV-encoded circRNAs especially calls into question many previously characterized pro- and anti-viral factors that may now have newly defined roles in circRNA biogenesis and regulation or even as circRNAs themselves. One example of this is the protein adenosine deaminase acting on RNA 1 (ADAR1), a recently identified pro-viral inhibitor of RLR-dependent immune signaling, is also inhibitory to circRNA biogenesis, which could in-turn impact KSHV fitness [48]. Furthermore, given their abundance and enhanced stability, several groups have proposed the use of KSHV circRNA as promising tumor biomarkers [49]. An even more exciting front can also be found with the identification of human-derived circRNAs during de-novo infection of human umbilical vein endothelial cells (HUVECs) that appeared to have anti-viral properties [41]. The prevalence of these anti-viral circRNAs in the context of DNA and RNA virus infection foretells of an interesting angle of how post-transcriptional RNA configuration and associated modifications could account for an entirely new face of the innate immune response. As with many discoveries in molecular biology, pioneering our understanding of viral circRNAs in the context of KSHV infection will undoubtedly propel forward the evolutionary implications of this novel virus-host interface. Furthermore, of course, by understanding the mechanisms that govern back-splicing of herpesviral genomes, we will undoubtedly discover novel pathways of cellular splicing that embody unexplored circuits of post-transcriptional gene regulation. 

### 2.2. Nuclear Export

G-Quadruplexes. Recently G-quadruplexes (G4s) have gained a lot of attention. These secondary DNA or RNA structures arise from nucleic acids that are rich in guanine residues and result in G-quartets forming from Hoogsteen hydrogen bonding stack upon one another. Multiple proteins are required to help form or unwind these structures [50]. G4s have gained some notice for their control over DNA/RNA metabolism. DsDNA viruses have been found to be particularly enriched with putative G4-forming sequences (PQS) [51], and KSHV LANA gene was found to form G4s within its own mRNA [52]. It was demonstrated that LANA protein itself binds to the G4 regions of its own mRNA to prohibit its export to the cytoplasm and thus help control its expression. Moreover, mass spectrometry revealed that LANA G4s also recruit hnRNPA1, a host protein known to destabilize G4s. This reveals an interesting feedback loop whereby low LANA protein levels influence how hnRNPA1 can bind LANA mRNA, resulting in the destabilization of its internal G4s and allowing nuclear export. Once in the cytoplasm, LANA mRNA is translated and then translocate back to the nucleus, and as the concertation of LANA in the nucleus becomes sufficient, LANA displaces hnRNPA1 by binding its own mRNA, preventing export and overall lowering its expression. Although historically KSHV transcripts are seen to be similar to host mRNA as they are processed and exported from the nucleus, we are now finding novel mechanisms relating to structure that allow KSHV to fine-tune its gene expression even further.

### 2.3. Nuclear Decay 

ORF57 and nuclear RNAi-defective 2 (NRDE2). RNA quality control (QC) pathways play a crucial role in the regulation of gene expression and RNA fate. Nuclear RNA QC pathways monitor the presence of improperly matured RNA and prevent their export to the cytoplasm. Among these QC pathways, two play a significant role in controlling the longevity of nuclear transcripts. These pathways are the PABPN1 and PAPα/γ-mediated RNA decay (PPD) pathway and the cap-binding complex (CBC) in association with the nuclear exosome targeting (NEXT) complex, known together as the CBCN-mediated RNA decay pathway. These pathways act on the decay of polyadenylated, short, and intronless transcripts, such as improperly spliced pre-mRNA that do not meet the requirements to become fully processed mature mRNA. These pathways also oversee the regulation of promoter upstream transcripts (PROMPTs), [53] short intronless polyadenylated ncRNA that share striking similarities with KSHV mRNA. Because of these similarities, KSHV mRNAs were found to be often targeted by these host nuclear RNA QC pathways for degradation [54]. Co-evolution between KSHV and its host has led to the rise of mechanisms that allow KSHV transcripts to escape these pathways. Maybe more surprising, it was recently shown that KSHV also manipulates these host RNA nuclear QC pathways for its own benefit for the purpose of temporal regulation. One particular viral protein, ORF57, a lytic early gene that is active in a diverse range of functions related to KSHV mRNA, appears to be crucial in co-opting these QC pathways [54,55,56]. ORF57 has homologs throughout the herpesviral family: EB2 in EBV, UL69 in HCMV, and ICP27 in HSV-1, however, the function and expression of these homologs vary [56]. As such, ORF57 has been associated with a wide variety of roles ranging from being a viral splicing factor to controlling translation of viral transcripts [56]. Recently it has come to light that ORF57 plays a significant role in protecting KSHV viral transcripts from the PPD and CBCN nuclear decay pathways. Previous research had shown that ORF57 provides stability and protection of PAN RNA through recruitment of the host ALYREF to the PAN poly(A) tail [57,58]. ALYREF is a nuclear export factor that is part of the Transcriptional Export complex (TREX), which is required for the proper export of cellular transcripts from the nucleus [59]. This would suggest that protection from nuclear decay pathways by ORF57 would involve the export of RNA as ALYREF is vital to the RNA export complex. However, work by Stubbs et al. showed that PAN stabilization by ORF57 is completely independent of the nuclear export activity of ALYREF [57]. This raises the question of how ALYREF is providing protection to this RNA as this process is export-independent. Could ALYREF be recruiting a factor that disrupts NEXT and PPD decay pathways? Or is there some form of binding competition involved where ALYREF out-competes other factors to act as a binding partner in this complex, leading to the inability of NEXT or PPD components from binding and how ORF57 is mediating transcript protection through the recruitment of ALYREF?

To answer this question, Ruiz and colleagues explored the involvement of ORF57 and ALYREF in connection to the CBCN and PPD nuclear decay pathways. The authors knocked down various components of these host RNA QC pathways and observed the effects on mutant of PAN that lacks the cis-acting nuclear retention element (PAN∆ENE) and lytic transcript ORF59 [54,55]. In the first set of experiments, they discovered that depletion of the protein ARS2—a key factor in both decay pathways—led to stabilization of both PAN∆ENE and ORF59 in the absence of ORF57. ARS2 contributes to these decay pathways by recruiting an RNA helicase protein MTR4 (hMTR4) and an exosome cofactor [60]. Knockdown of hMTR4 was able to restore transcript levels of both PAN∆ENE and ORF59 in absence of ORF57, indicating that ORF57 protects transcripts from hMTR4-mediated decay. Previous work by Fan et al. showed that ALYREF and hMTR4 compete for interactions with cellular pre-mRNAs: ALYREF is recruited to mature mRNAs through interactions with ARS2 to stabilize and export transcripts, whereas hMTR4 binds to ARS2 on transcripts designated for degradation [61]. Through this work, Ruiz and colleagues developed a model in which ORF57 binds to viral transcripts and recruits ALYREF, which outcompetes hMTR4 for binding of ARS2, effectively protecting the transcripts from host RNA QC decay pathways. This pathway occurs without export of the viral transcripts even though the mechanisms exploited are major export factors. It is possible that some other viral factors maintain nuclear localization of these ALYREF bound factors. Maybe further ORF57 interactions lead to eventual export of viral transcripts through the removal of ALYREF and the recruitment of the TREX complex. As it has been previously shown that depletion of Aly, a homolog for ALYREF (ALY/REF), only leads to a slight decrease in nuclear export of viral transcripts [62], which suggests that ALYREF is not necessary for the export of intronless viral mRNA by TREX. Manipulation of ALYREF might thus play a wide range of roles surrounding nuclear viral transcripts, including protection from exosome-mediated decay pathways and, potentially, some role in transcript export.

Despite the observation that ORF57 is capable of protecting viral transcripts form nuclear RNA QC decay pathways, it was observed that a small subset of transcripts still underwent PPD and CBCN modulated decay even in the presence of ORF57 [54,55]. Using RNA-seq after knockdown of ARS2 and PAPα/γ, it was discovered that several viral delayed early and late transcripts were upregulated [55]. These transcripts arise from cryptic transcription where transcripts arise out of normal timelines due to intragenic promoters. It was determined that the expression of these cryptically expressed late genes was controlled for by the PPD RNA QC pathway. Outside of the lytic late phase, hMTR4 and ZCCHC7, a zinc finger containing protein that is part of the TREX complex, binds to these transcripts and exosome-mediated decay stops accumulation of late genes. However, these same transcripts are protected from the PPD pathway and accumulate to appropriate levels. This temporal regulation was linked to nuclear speckles and the host nuclear RNAi-defective 2 (NRDE2) protein. NRDE2 is a negative regulator of exosomes through the formation of complexes with MTR4, which leads to inhibition of exosome-mediated degradation [63]. During late replication, the viral replication compartment merges with nuclear speckles where NRDE2 resides, leading to the formation of NRDE2 interactions with MTR4, which prevents the assembly of the PPD-mediated exosome complex [55]. This protection is mediated by NRDE2 recruitment to mRNA, which inhibits the binding of MTR4, much like the ORF57 regulation of nuclear decay pathways.

Through these mechanisms, KSHV can hijack and manipulate host nuclear RNA regulatory pathways to promote infection. Both systems exploit host RNA QC pathways primarily through the manipulation of the mRNA export adaptor ALYREF. While the NRDE2 pathway does not directly require ORF57, interactions surrounding this event may also include ORF57, as it is vital for the recruitment of ALYREF to specific transcripts and plays a major role in splicing within KSHV replication compartments merged with nuclear speckles where it colocalizes with splicing factors [64]. One aspect of these processes that is interesting is that ALYREF is primarily an export adaptor used to move nuclear transcripts to the cytoplasm, but all these events are independent of transport. This would suggest that some other event is blocking TREX-mediated export of these transcripts, or, especially in the case of late KSHV transcripts, these events may precede the formation of the TREX complex. Similar interactions were observed in the ORF57 homolog ICP27, which recruits ALYREF to HSV-1 transcription sites without recruiting TAP/NXF1 to form the Transcriptional Export complex (TREX), and ALYREF is not required for viral transcript export [65]. Perhaps once late KSHV transcripts have to be protected from MTR4-mediated exosome degradation by NRDE2 some other factor disassociates NRDE2 and allows formation of the TREX complex linked to the presence of ALYREF on these transcripts. Since PAN readily binds both ALYREF and PABPC1, both of which play a role in mRNA export while being unrelated to export of PAN RNA, it would also be interesting to see if there is some link between the export of these late proteins and PAN RNA. Maybe there may be interactions between PAN RNA and late transcripts bound with ALYREF that may lead to nuclear export? The link between viral transcript stability and protection to nuclear export accessory proteins may just be a methodology for tricking hosts systems and recruiting other proteins, or maybe there is some link between these protection systems and the eventual export of viral transcripts.

## 3. Cytoplasmic Regulation of RNA during KSHV Infection

**Figure 2:**
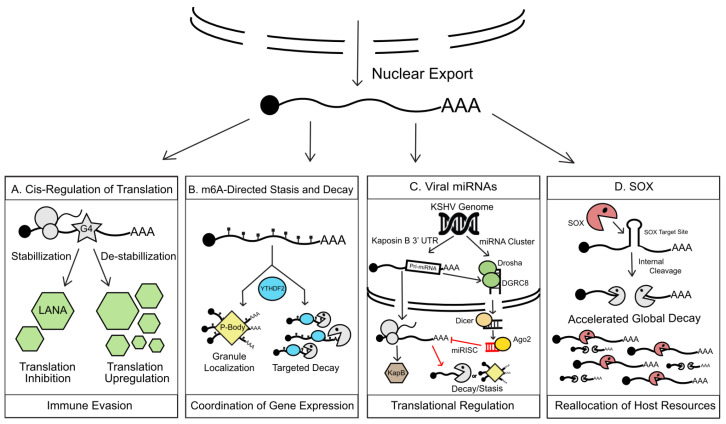
KSHV manipulation of cytoplasmic RNA regulation. (**A**) G4s found within cytoplasmic LANA transcripts, when stabilized, inhibit LANA translation, which in turn reduces LANA-triggered antigenic presentation aiding in immune evasion during KSHV latency. (**B**). The fate of m^6^a modified viral transcripts in the cytoplasm is modulated by m^6^a readers. These readers, including YTHDF2 (YTH N6-Methyladenosine RNA Binding Protein 2), orchestrate silencing and/or degradation of modified mRNAs by driving their localization to cytoplasmic RNA granules such as P-bodies or recruitment of host exonucleases. Co-opting of this m^6^a machinery allows KSHV an unprecedented level of control over temporal viral gene expression. (**C**) KSHV encodes a large repertoire of viral miRNA. Taking advantage of the cellular miRNA silencing complex (RISC), viral miRNAs regulate translation of viral and host transcripts in trans by redirecting their localization to RNA granules for homeostasis or immediately triggering RNA decay. As a form of cis-regulation of a viral transcript, KSHV also co-opts cellular miRNA biogenesis machinery, namely DROSHA, to restrict the over-production of KapB during latency, which contributes to cell survival during latency and temporal regulation of KapB. (**D**) To swiftly seize control of the cell during lytic reactivation, KSHV encodes a viral endonuclease, SOX, which triggers a global RNA decay event. SOX selectively cleaves host and viral transcripts that bear conserved SOX recognition sites, rending transcripts susceptible to decay by cellular exonucleases. This massive degradation event releases host resources such as the translation machinery and RNA-binding proteins for reallocation to viral gene expression.

During KSHV infection, as mRNAs enter the cytoplasm they are subjected to an even greater repertoire of cellular and viral factors that dictate whether they are translated, stored, or degraded. KSHV co-evolution with its human host has resulted in a wide breadth of viral intrusion into cytoplasmic RNA surveillance and mimicking of RNA features such as RNA interference or RNA structural elements. These processes emerge as crucial to suppress unwanted mRNA translation in favor of both latency maintenance and lytic viral replication (Figure 2).

### 3.1. mRNA Translation

G-quadruplexes. As we previously discussed, G4s within LANA transcript are important to regulate LANA export from the nucleus, but it was also suggested that these G4s also play a role in the cytoplasmic regulation of LANA. It was discovered that when LANA G-quadruplex is stabilized, LANA translation was inhibited and, therefore, protein expression was reduced, despite normal mRNA synthesis. Furthermore, when LANA mRNA was mutated to disrupt the G4 formation, it was revealed that LANA translation was increased, further supporting G4s as a translation regulator of LANA. LANA has been shown to modulate antigen presentation by interacting with components of the antigen presentation [66,67]. G4s are now thought to be another way KSHV dampens immune responses by reducing translation of LANA1 and, therefore, this reduces antigen presentation [52]. Together it can be seen that G4s are being used as a regulatory element between host and virus. 

### 3.2. RNA Decay

M^6^A. As previously mentioned, m^6^A modifications influence many aspects of an mRNA life. In the cytoplasm, m^6^A readers have been shown to affect the stability, localization, and translation of mRNAs. One such reader that has come up as an important regulator of the cytoplasmic viral mRNA pool is YTHDF2. This reader binds directly to m^6^A-modified mRNA and promotes their degradation by localizing them to RNA granules known as processing bodies (P-bodies) [68,69]. Hesser et al. found that depleting YTHDF2 in the KSHV positive cell line ISLK.219 drastically restricts KSHV lytic cycle and subsequent virion production. Notably, it was uncovered that this mechanism is mediated by restricting the amount of RTA production, which suggests a pro-viral role of m^6^A in promoting lytic reactivation [23]. In stark contrast, another study in the iSLK cells published by Tan et al. suggested that depletion of YTHDF2 instead results in increased KSHV replication and thus appears to posit an anti-viral role for this m^6^A reader [19]. On the other hand, Hesser et al. also noted that contrary to their iSLK results, in another KSHV positive cell line, TREX-BCBL1s, depletion of YTHDF2 resulted in an accumulation of RTA, which suggests that m^6^A in this case would have an antiviral role [23]. It is still unclear why various studies and cell lines appear to have contradictory results, but it would suggest that m^6^A deposition and/or m^6^A-mediated functions are highly dynamic. One possibility is that m^6^A regulation is dependent on temporal factors and that fine-tuning of viral mRNA stability could be a requirement for proper progression of the viral life cycle. Furthermore, mapping of m^6^A sites within viral mRNA revealed that despite some differences, many of the m^6^A peaks are consistent across multiple cell types: the same transcripts overall are methylated but the position of this methylation along the transcript can vary. This suggests that the viral mRNAs are not necessarily interfacing with host m^6^A methyltransferase machinery differently between cell types but instead the contradictory results further enforce the idea that m^6^A deposition is dynamic and does not always occur on every possible m^6^A motif. Localization of the m^6^A mark can be tied to the rate of RNA Pol II elongation where a slow elongation leads to enhanced m^6^A modification and eventually decrease of translation efficiency [15,16]. It is thus possible that the speed of RNA pol II–dependent KSHV transcription will similarly impact m^6^A deposition and thus be highly context-dependent, possibly explaining this variability. Understanding where and what m^6^A depositions mean on an mRNA may help in understanding their effects on KSHV replication. 

KSHV miRNA. Having passed an extensive panel of nuclear checkpoints, cytoplasmic mRNAs fall under an equally vast network of regulatory oversight evolved to control the terminal stages of their fate from translation to decay. One form of cytoplasmic RNA surveillance are small non-coding RNAs known as microRNAs (miRNA). miRNAs are short (19–23 nt), single-stranded RNAs that coordinate with cellular RNA silencing factors to dictate the rate of translation and longevity of target transcripts. Mechanistically, miRNAs often target complementary sites within the 3′ UTR or occasionally the coding region of their cognate mRNA. Binding specificity of miRNAs to these sites typically relies on a minimum seed sequence match (2–7 nt), but this requirement has been challenged by several recent reports [70]. Once bound, miRNAs associated with the cellular silencing machinery will either block the recruitment of the translation initiation complex or destabilize the RNA triggering RNA decay. miRNAs, being short sequence fragments, also lack markers that identify them as “self” making them non-immunogenic. However, this same feature represents a significant Achilles heel for the cell, allowing virally encoded miRNAs ease of access to microRNA-induced silencing pathways. Taking advantage of this, KSHV encodes a highly conserved array of 12 precursor miRNAs that give rise to over 25 mature viral miRNAs [71,72]. Most of these miRNAs are expressed from a single large genomic cluster located between the Kaposin locus and ORF71 while two are located within the Kaposin locus itself. Both non-immunogenic and profound in their capacity for post-transcriptional RNA regulation, the viral miRNAs are critical to the progression of the KSHV life cycle, reinforcing latency maintenance, productive lytic infection, and even KSHV carcinogenesis [73,74,75,76,77]. Here, we will discuss how KSHV-encoded miRNAs regulate the fate of cytoplasmic viral RNAs and the contribution of these pathways toward temporal regulation of KSHV gene expression. 

Stringent control over the switch between the latent and lytic stages of KSHV infection is critical for a successful persistent infection and timely coordination of viral replication. Most KSHV-encoded miRNAs are predominantly expressed during the latent phase of infection except for those derived from the Kaposin locus, which is highly expressed during the lytic phase. These expression patterns are evident across most KS/MCD derived tissues and cell lines albeit with inconsistency between expression levels of the miRNAs themselves [78]. Therefore, many viral miRNAs are inherently dedicated to altering the gene expression environment in support of latency maintenance and restriction of the host anti-viral response. KSHV miRNAs meet both objectives by targeting the mature mRNA of the major lytic transactivator, KSHV RTA (ORF50). Demonstrated in a variety of KSHV latent cell lines, miR-K12-7 and miR-K12-9 both directly restrict RTA via binding of its 3′ UTR and recruitment of the silencing machinery [73,79,80]. Several other viral miRNAs also indirectly regulate RTA expression by targeting host genes that regulate its transcription such as MYB and nuclear factor 1B (NFIB) or by targeting factors that modulate the RTA promoter such as RB Transcriptional Corepressor Like 2 (RBL2) and Bcl-2-associated factor-1 (BCLAF1) [73,79,81,82]. While several other mechanisms are used to restrict lytic gene expression during latency, it is generally agreed that miRNA suppression of RTA represents an additional “failsafe” to ensure the timely expression of the lytic gene cascade. The extent of this post-transcriptional regulation by miRNAs of RTA mirrors the lengths to which KSHV has evolved to maintain a strict control over the lytic switch, and surprisingly represents only a sliver of RNA surveillance involved in maintaining this balance. 

While RTA represents the most direct example of how KSHV-encoded miRNAs regulate the viral life cycle, only a handful of groups have identified other miRNA targets of viral origin. Aside from RTA, these earlier studies identified a few viral gene targets including ORF56, ORF57, and viral interleukin-6 (vIL-6) [73,80,83]. Further identification of miRNA-targeted viral transcripts beyond these was initially hindered due to the complexity of viral 3′UTRs for the majority of the KSHV transcriptome, often since many are comprised of smaller viral ORFs. Addressing these obscure areas of miRNA study, several groups have expertly mapped the 3′ UTRs of the remaining KSHV genes (accounting for 80% of KSHV coding capacity) and identified novel miRNA-targeting sequences therein [8,84,85,86]. Of these 3′ UTRs, Bai and colleagues found 28 that were directly targeted by KSHV miRNAs with 11 of these located within bi- and polycistronic transcripts including ORF30-33 and ORF71-73 [86]. These observations not only expanded our understanding of how KSHV expands its coding capacity utilizing multiple ORFs but also demonstrated that the KSHV-encoded miRNAs have a far more expansive role to play in the regulation of lytic viral replication. 

While miRNA regulation of KSHV genes is typically discussed from the perspective of trans-regulation, pre-miRNAs that give rise to mature viral miRNA can also regulate viral gene expression in cis. In canonical miRNA biogenesis, primary-miRNAs (pri-miRNA) hairpins are transcribed and processed by the nuclear microprocessor complex comprised of DiGeorge Syndrome Critical Region 8 (DGCR8) and the ribonuclease III enzyme, Drosha into long duplex RNA fragments known as pre-miRNAs. These RNA duplexes are then exported into the cytoplasm where they give rise to mature miRNAs. Two pre-miRNAs, miR-K12 and miR-K10, located apart from the remaining 23, are encoded by 3′UTR of the coding gene Kaposin B (KapB). Many KSHV genes are divided into either latency or lytic gene families based on their expression patterns throughout viral infection. However, several KSHV transcripts, including KapB, are constitutively expressed at low levels during latency and highly expressed following lytic reactivation [87]. A currently expanding list of functions has been attributed to KapB ranging from induction of cellular cytokines to catabolism of RNA granules [88,89]. Of note is the apparent cytotoxicity induced by the overexpression of KapB in PEL cells, suggesting that its restricted expression during latency is required to enhance cell survival, driving the need for an active level of post-transcriptional regulation [87]. With a profound breadth of functions and its negative impact toward latency maintenance, regulation of KapB throughout the latent and lytic phase is critical to ensuring viral fitness. Several lines of evidence have demonstrated that processing of nascent RNAs that are both coding mRNA and contain sequences for pri-miRNAs inevitably are degraded once cleaved by Drosha for pre-miRNA biogenesis [90,91,92]. Therefore, these binary RNAs are forced to meet one of two fates based on the signaling that drives the maturation of miRNAs. Based on this, Lin et. al. hypothesized that the pre-miRNAs for miRs K12-10 and K12-12, found within the KapB 3′ UTR, function as negative cis-regulatory RNA elements that dictate the fate of the KapB nascent pre-mRNA [93]. Through a series of mutagenesis experiments, they demonstrated disruption of the pre-miRNA structures or lack thereof significantly enhanced KapB protein levels and confirmed that the mature miRNAs themselves did not negatively regulate KapB in trans. Furthermore, this cis-regulation was mediated by the cellular microprocessor machinery itself, namely Drosha, and that KSHV lytic infection restricted Drosha expression drastically to then in-turn upregulate KapB expression. Collectively, these observations extended the role of Drosha from solely miRNA biogenesis to the active regulation of viral gene expression, as well as establishing a newfound level of post-transcriptional regulation that allows KSHV to take advantage of the cellular microprocessor to regulate its own life cycle. 

This binary fate of the KapB mRNA highlights the interconnectedness between post-transcriptional regulation of viral mRNA and viral miRNA biogenesis. It would be exciting to see if pre-miRNAs of other unrelated DNA tumor viruses, especially those encoded in the 3′ UTRs or exons of coding transcripts, have also been evolved to serve as cis-regulatory factors in the regulation of viral gene expression. Along the same vein, adaptation of the microprocessor machinery by KSHV opens a number of avenues of investigation including studies of viral manipulation of miRNA processing beyond Drosha, the regulation of Drosha in respect to cellular miRNA biogenesis during infection, and the host mRNAs they regulate therein. Much work is still necessary to fully uncover the depth to which miRNAs influence the fate of viral RNAs and the physiological consequences of this activity. Several studies have reported functions for miRNAs far beyond this scope, especially with reports describing their incorporation into extracellular vesicles and even KSHV virions for signaling in a paracrine like manner [94,95]. With these findings in mind, several questions remain regarding what cellular and viral factors drive the expression of miRNAs and why their expression levels vary drastically between KSHV life cycle stages and tissue-derived samples. Furthermore, new lines of research have arisen looking into cellular factors directly linked to miRNA surveillance such as the influence of KSHV infection over RNA phase-separated P-bodies [96,97]. P-bodies have been directly linked to the active surveillance of miRNAs and have been proposed to enhance their silencing activities [98]. Given the drastic difference in P-body manipulation throughout the viral life cycle and how KSHV’s ORF57 antagonizes P-body assembly, it would be interesting to learn how these dynamics influence viral miRNA activity and how this relationship impacts the active surveillance of viral mRNAs that are targeted by viral miRNAs. 

SOX. Similarly to ORF57 that regulates RNA decay in the cytoplasm, KSHV also has a major RNA decay regulator in the cytoplasm, SOX (ORF37). This protein is expressed as a delayed early gene during lytic reactivation and is responsible for the host shutoff event, which results in decreased host gene expression [99,100]. During this event, SOX induces widespread mRNA decay by targeting host and viral mRNA via an UGAAG degenerate RNA motif located along a rather stable stem-loop structure [101,102]. SOX’s effect on the transcriptome is extensive and was estimated that close to 70% of all mRNA are targeted for degradation upon SOX expression and, in fact, most human and viral transcripts contained at least one sequence fitting this SOX targeting motif [101]. Once SOX recognizes its targeted transcript it creates an endonucleolytic nick on mature mRNAs. This endonucleolytic cleavage is the hallmark for the activation of the host RNA decay machinery, in particular the exonucleases Xrn1 and Dis3L2 that rapidly degrade the newly exposed 5′ and 3′ fragments created by SOX-mediated cut [103]. This rapid and widespread process quickly reduces the amount of cytoplasmic mRNA while at the same time releasing many RNA-binding factors from these now degraded mRNA [104]. These factors suddenly without a target mRNA to bind to are now free to shuttle in the cell and this was shown to trigger a feedback mechanism that, in response to this massive RNA decay event, halts transcription in the nucleus [105,106]. This feedback loop was revealed to have an important pro-viral function as only host genes are affected that favor recruitment and elongation of RNAPII on viral promoters [106]. SOX-mediated RNA decay thus helps free up the translational machinery, which rapidly takes up the only mRNA available: KSHV-encoded mRNA. Although SOX-induced host shutoff affects a majority of cellular mRNA transcripts, there have been studies showing that a small number of transcripts robustly escape decay [107,108,109,110,111,112]. It is still unclear by what mechanisms these escapees avoid being either targeted or degraded by SOX. Some recent work uncovered an RNA element named SOX-Resistant Element (SRE) in the 3′ UTR of certain mRNAs preventing SOX cleavage [111,112]. While the presence of this RNA element is crucial to protect select transcript from degradation, the exact mechanism of its broad resistance is still not understood. In light of the extensive hijacking by KSHV of its host RNA regulation pathways described in this review, it would be interesting to investigate whether the SRE interfaces with any of the mechanisms highlighted here.

The continued research into KSHV’s pervasive use of cellular regulatory pathways has revealed new insights into how KSHV enhances its infection as well as new aspects of cellular RNA fate. Although this research as given more insights into KSHV regulation, more questions remain. New roles for m^6^A in the context of KSHV have been elucidated, however, there are conflicting roles depending on cell type and the reason for this remains to be discovered. Similarly, new research has been done into KSHV miRNAs now leaves questions regarding how the host monitors these miRNAs and how the dynamics of P-bodies affect their function as well. KSHV’s use of the G4 structural element is fascinating and makes one wonder if there are more transcripts that use G4s or other structural elements to regulate their translation efficiency. Finally, although a lot of research is done into viral endonucleases like KSHV SOX, there are still mysteries surrounding the mechanisms of targeting and escape of transcripts. Answering these outstanding questions will reveal new avenues of KSHV cytoplasmic regulation of RNA in the cell. 

## 4. Conclusions

The past two decades have uncovered exciting new ways by which KSHV extensively regulates both its own and its host’s RNA during infection. This emphasizes KSHV large investment in the everlasting battle with its host over the gene expression cascade. Viruses have historically been good models to gain a deeper understanding of basic cellular mechanisms, and by studying how KSHV manipulates RNA fate, we continue to discover novel aspects of the gene expression regulation. It also underlines the critical role of the RNA fate equilibrium and how disrupting that balance can have far-reaching consequences. Currently, there is still much to study and understand about what governs these mechanisms. In particular, we are only starting to appreciate the contribution of G-Quadraplexes or m^6^A on KSHV biology. The extent of factors that can interact with these structural and chemical modifications is by no means a complete list. Finding and characterizing these factors will greatly expand our knowledge of RNA fate as well as increase our understanding of KSHV host takeover. There are also still over a hundred different RNA modifications and post-transcriptional RNA editing that have been documented like inosine (I), pseudouridine (Y), and 2′-O-methyladenosine (Am). Although m^6^A has been studied in the context of KSHV, these other RNA editing strategies may be used by the virus in order to regulate cellular and viral RNAs [8]. Given the recent discovery of KSHV circRNAs, future work will help us understand the nature of these novel regulatory RNAs and their effect on KHSV infection. Excitingly, the discovery of their presence into virions opens a whole new avenue of possibilities. Previous research into circRNAs have helped redefine circRNAs role in RNA fate, further characterization of circRNAs in the context of KSHV infection will undoubtedly expand the field of KSHV post-transcriptional regulation. Another area that remains elusive is how some mRNA can escape the considerable energy that KSHV deploys to trigger their decay. As described in this review, KSHV unfolds a vast network of stratagems to have a tight control over RNA fate—both on viral but especially on host mRNA. However, we know of several host transcripts that can bypass all these mechanisms and are robustly expressed during infection. As we are only scratching the surface of structural and chemical alterations and how these processes have the capability to recruit many trans-acting factors, it is possible that presence of G4s or m^6^A modification could also contribute to the host side of the viral-host battle. By understanding these processes further, we continue to better appreciate how the tenuous and important the balance of RNA fate is.
